# Subtenon triamcinolone as an adjuvant in mitomycin-C-enhanced trabeculectomy in non-inflammatory glaucomas: A randomized clinical trial

**DOI:** 10.1371/journal.pone.0268623

**Published:** 2022-05-26

**Authors:** Diego T. Dias, Izabela Almeida, Michele Ushida, Flavio S. Lopes, Fábio N. Kanadani, Carolina P. B. Gracitelli, Tiago S. Prata

**Affiliations:** 1 Department of Ophthalmology, Federal University of São Paulo, São Paulo, São Paulo, Brazil; 2 Glaucoma Department, Hospital de Olhos de Sergipe, Aracaju, Sergipe, Brazil; 3 Glaucoma Unit, Hospital Medicina dos Olhos, Osasco, São Paulo, Brazil; 4 Department of Ophthalmology, Hospital Oftalmológico de Sorocaba, Sorocaba, São Paulo, Brazil; University of Pavia, ITALY

## Abstract

This unicentric randomized clinical trial was designed to compare the surgical outcomes of mitomycin C-enhanced trabeculectomy (MMC-TRAB) with and without subtenon triamcinolone acetonide (TAAC) injection in patients with non-inflammatory glaucomas. This trial is registered at the Brazilian Registry of Clinical Trials (ReBEC) under the register number RBR-53f8nh. Consecutive non-inflammatory glaucoma patients requiring surgical intervention were randomized into two groups. In the control group, eyes underwent standard MMC-TRAB, while in the intervention group, besides the standard MMC-TRAB, these eyes also received a subtenon TAAC injection (4mg) close to the bleb site at the end of the surgery. The main outcomes of the study were surgical success rates, intraocular pressure (IOP) and number of medications at all timepoints. Success was defined as IOP ≤ 15 mmHg and subdivided in complete or qualified according to the need of medication. A total of 75 eyes of 63 different patients were included (intervention group = 39 eyes; control group = 36 eyes). There was no difference between groups at baseline (p>0.11). Multivariable regression analysis indicated that IOP levels were significantly lower in the intervention group at 18 and 24 months of follow-up when number of medications was considered as a covariate (P<0.001). Complete success rates were higher in the intervention group at 06 (90.9% vs 68.7%; p = 0.03), 12 (87.2% vs 66.7%; p = 0.02) and 18 months (87.2% vs 66.7%; p = 0.02). Additionally, although success rates at 24 months were higher in the intervention group (82.0% vs 66.7%; p = 0.09), this difference did not reach statistical significance. Qualified success rates did not significantly differ between groups at all timepoints. In conclusion, this study found significantly lower IOPs levels at 18 and 24 months of follow-up and higher complete success rates until 18 months of follow-up, with the use of subtenon TAAC as an adjuvant to standard MMC-TRABs in non-inflammatory glaucoma patients.

## Introduction

Glaucoma is the main cause of irreversible blindness worldwide [1]. It is estimated that approximately 64.3 million people between 40 and 80 years old are currently affected by the disease, and this number may rise up to 76 million, in 2020, and 111.8 million, in 2040 [2]. Glaucoma treatment is usually started with hypotensive eyedrops, while incisional surgeries are often employed in medically uncontrolled cases, which did not respond to maximum tolerated therapy [3, 4].

For more than 20 years, trabeculectomy (TRAB) has been the most widely employed incisional surgical technique for glaucoma treatment worldwide [5]. Nonetheless, surgical success depends on controlled inhibition of the wound healing process [6, 7]. In this context, surgical failure is most frequently associated with excessive scar tissue formation in the subtenon space which precludes adequate aqueous outflow through the created fistula [8, 9]. Modulation of wound healing measures, such as the intraoperative use of antifibrotic drugs (the most widely employed is mitomycin C–MMC) [10–12] and postoperative topical corticosteroids [13–15] are, therefore, routinely employed in glaucoma surgery, as they improve success rates in TRABs.

Corticosteroid eyedrops have been for a long time paramount for wound healing control as a postoperative therapy in TRABs. Additionally, subtenon injection of triamcinolone acetonide (TAAC) is not only a safe and effective ophthalmic procedure, but also a well-established therapeutic option for several ophthalmic conditions (such as posterior uveitis [16], diabetic macular edema [17] and macular edema secondary to vascular occlusions [18]). With that in mind, one might hypothesize that a deposit corticosteroid formulation, such as TAAC, might increase its postoperative bioavailability, and possibly improve wound healing control and surgical outcomes in TRABs. The purpose of this randomized clinical trial was to assess the adjuvant effect of subtenon TAAC injection in surgical outcomes of primary TRABs in individuals with non-inflammatory glaucomas.

## Materials and methods

This protocol adhered to the tenets of the declaration of Helsinki and was approved by the ethics committee and the institutional review board of the Federal University of Sao Paulo. This trial is also registered at the Brazilian Registry of Clinical Trials (ReBEC) under the register number RBR-53f8nh. The full protocol can be assessed at http://www.ensaiosclinicos.gov.br/rg/RBR-53f8nh. All patients provided written informed consent prior to enrollment and examination.

### Study design

This was an unicentric, randomized for an intended 1:1 allocation ratio, parallel-group, unmasked clinical trial, designed to assess the adjuvant effect of subtenon TAAC in surgical outcomes of primary TRABs in individuals with non-inflammatory glaucomas.

### Participants

We enrolled consecutive glaucomatous patients with medically uncontrolled non-inflammatory glaucomas and indication for TRAB attending to the Glaucoma Sector of Hospital Medicina dos Olhos (Sao Paulo, Brazil). Whenever both eyes of the same patient were considered eligible for surgery, each eye was considered as an independent unit, meaning that the same patient could have both eyes allocated to the same group or one eye to each group.

Glaucoma was defined as the presence of glaucomatous optic neuropathy (GON). We defined GON as cup-to-disc ratio >0.6, asymmetry between eyes ≥0.2, presence of localized defects of the retinal nerve fiber layer, and/ or neuroretinal rim in the absence of any other anomalies that could explain such findings. Non-inflammatory glaucoma was defined as glaucoma not associated with ocular inflammation (neovascular or glaucoma associated with uveitis). Additionally, characteristic glaucomatous visual field (VF) defect was defined as glaucoma hemifield test results outside normal limits and the presence of at least 3 contiguous test points within the same hemifield on the pattern deviation plot at P<1%, with at least 1 at P<0.5%, excluding points on the edge of the field or those directly above and below the blind spot [19, 20]. Finally, a patient was considered to be medically uncontrolled based on anatomical and/or functional progression (detected by retinography or perimetry, respectively) or if IOP values were above the target range defined by the attending physician based on VF, optic nerve examination, age and risk factors, in accordance with European guidelines [21].

Patients were excluded if they met one of the following criteria: diagnosis of inflammatory glaucoma (neovascular or glaucoma associated with uveitis); diagnosis of medically uncontrolled non-inflammatory glaucoma associated with clinically relevant cataracts, and indication of combined surgery (phacoemulsification and TRAB); and previous intraocular surgery, except for uncomplicated phacoemulsification or laser surgery if performed more than 6 months prior to glaucoma surgery.

### Procedures and surgical technique

All participants underwent a comprehensive ophthalmological evaluation, including best-corrected visual acuity, slit-lamp biomicroscopy, IOP measurement, gonioscopy, dilated fundoscopy, VF testing (24–2 Swedish interactive threshold algorithm, Humphrey Field Analyzer II; Carl Zeiss Meditec, Inc., Dublin, CA), optic disc stereophotographs, and color/ red-free fundus imaging. Clinical and ocular data including age, race, gender, IOP, central corneal thickness (CCT), VF mean deviation (MD), previous intraocular surgery and type of glaucoma were assessed at the baseline visit. Follow-up visits were scheduled at 1, 3, 6, 12, 18 and 24 months postoperatively. Additional follow-up visits were performed whenever needed.

After inclusion, patients were randomly enrolled in two groups by flip-coin technique, performed by the chief-nurse at the operating room immediately before surgery. In the control group, eyes underwent a standard MMC-enhanced TRAB (MMC-TRAB), while in the intervention group, besides the standard MMC-TRAB, these eyes also received a subtenon TAAC injection (4mg) close to the bleb site at the end of the surgery.

All surgeries were performed by 4 surgeons with previous glaucoma surgery experience (T.S.P., D.T.D., I.A., and M.U.), following a standard technique under peribulbar anesthesia and sedation. In order to maintain homogeneity of the surgical technique, T.S.P. was present in all of the procedures. Initially, a traction corneal suture was performed to allow better exposure of the surgical site. This was followed by incision of the conjunctiva and Tenon’s capsule 1-2mm posterior to the limbus and fornix-based subtenon dissection posteriorly in the superior quadrant, with concomitant hemostasis as needed. MMC at the concentration of 0.33 mg/ml was then applied for 3 minutes under the Tenon’s capsule using 3 separate soaked sponges. This was followed by dissection of a rectangular 4x2mm half-thickness scleral flap up to the clear cornea using a crescent knife, resection of an anterior trabecular block and peripheral iridectomy. The scleral flap was then sutured with two or three 10–0 nylon sutures, and the suture tension was adjusted to allow adequate flow of aqueous and at the same time maintain anterior chamber depth. Finally, the conjunctiva and Tenon’s capsule were closed with separate 10–0 nylon sutures. The only difference between groups was the subtenon TAAC injection (4 mg– 0.1 ml; Ophthalmos Pharmaceuticals) at the bleb site 7mm posterior to the limbus in the intervention group.

The postoperative eyedrop regimen followed the same protocol for both groups. All patients were treated postoperatively with topical antibiotic eyedrops (moxifloxacin) 4 times daily for a week, topical 1% atropine 2 times daily for 2 weeks and topical corticosteroid eyedrops (prednisolone 1%). The topical corticosteroids were initially applied every 1 hour and this dose was then tapered according to postoperative conjunctival inflammation. Additionally, globe massage and laser suture lysis were performed as needed by the attending physician and the introduction of hypotensive eyedrops was performed in a stepwise protocol [22] whenever the patient presented at two consecutive appointments with IOP higher than the preoperatively defined target range. Finally, when IOP control was not achieved with the reintroduction of medication, needling was performed, and, if IOP remained higher than the preoperatively defined target range at two consecutive appointments, hypotensive eyedrops were reintroduced in a stepwise manner. Globe massage was recommended as a home-based maneuver to be performed by the patient at the clinician’s discretion.

### Clinical outcomes and definitions of success

The main outcome of this clinical trial was difference in IOP between groups at the 24-month follow-up. Secondary outcomes were difference in postoperative success rates and number of hypotensive eyedrops between groups at all timepoints.

In accordance with the World Glaucoma Association Guidelines on Design and Reporting Clinical Trials [23], we defined success as IOP ≤ 15mmHg. Whenever success was achieved without the need for additional hypotensive medications, this was considered complete success. On the other hand, whenever the IOP-based criterion was achieved only after the introduction of hypotensive eyedrops, this was considered qualified success.

Additionally, surgical failure was defined as the presence of one of the following criteria: loss of light perception, IOP that does not fulfill the success criteria in 2 consecutive follow-up visits and IOP-lowering reoperations. Needling procedures or surgical approaches due to overfiltration (such as conjunctival restrictive sutures or choroidal drainage) during the first 6 months of follow-up were not considered surgical failures, since they were considered early postoperative-related maneuvers. Nonetheless, whenever these procedures were necessary after 6 months of follow-up, they were labeled as reoperations and, therefore, surgical failures.

Needling and reoperations rates and surgical complications, such as hyphema, hypotonic maculopathy, choroidal detachment and persistent bleb leakage were also evaluated at the same timepoints. In this context, a bleb leakage was considered to be persistent whenever it did not resolve spontaneously after 1 month with conservative medical management or required surgical reintervention [24].

### Sampling and statistical analysis

Considering the primary outcome of our study, we chose the magnitude of IOP reduction difference between groups at 24 months of follow-up as the main variable for sample size calculation. Based on the recommendations of the Guidelines on Design and Reporting of Glaucoma Surgical Trials, the trial’s sample size estimate was based on a desire to detect a specified, clinically relevant difference between the study surgery and the standard treatment in the primary efficacy outcome of the study. Therefore, for a sample power of 80% (β value of 0.20) and α value (type I error) of 0.05, we would need 29 patients in each group to detect an IOP difference of 3 mmHg between groups at the 2-year follow-up (assuming a standard deviation of 4 mmHg).

The analysis of our results were based on initial randomization (Intent to Treat Analysis). Descriptive analysis was used to present demographic and clinical data. D’Agostino-Pearson test was performed to determine whether data had normal distribution. Normally-distributed data were presented as mean and standard deviation, whether non normally-distributed data were presented as median and interquartile intervals. Regarding the comparison between groups, continuous data were compared using *t* test or Mann-Whitney test, depending on the data distribution. Fischer’s exact test or Chi-square test were performed to evaluate categorical variables whenever appropriate. Kaplan-Meier survival analysis was used to estimate and compare success rates between groups along the postoperative follow-up period. All included eyes were considered for the survival analysis. To control for dependencies between both eyes, we performed cox regression analysis with bootstrap resampling method for clustered data using 100 replications [25], adjusted for dependencies between both eyes. Finally, a multivariable analysis with random coefficients models was used to evaluate the effect of possible confounding factors on the relationship between type of intervention and IOP at all endpoints. Variables investigated included baseline IOP and number of medications. Computerized analysis was performed using commercially available softwares (MedCalc Inc., Mariakerke, Belgium; and Stata, version 17; StataCorp LP, College Station, TX.) and statistical significance was set at P<0.05.

## Results

**Fig 1:**
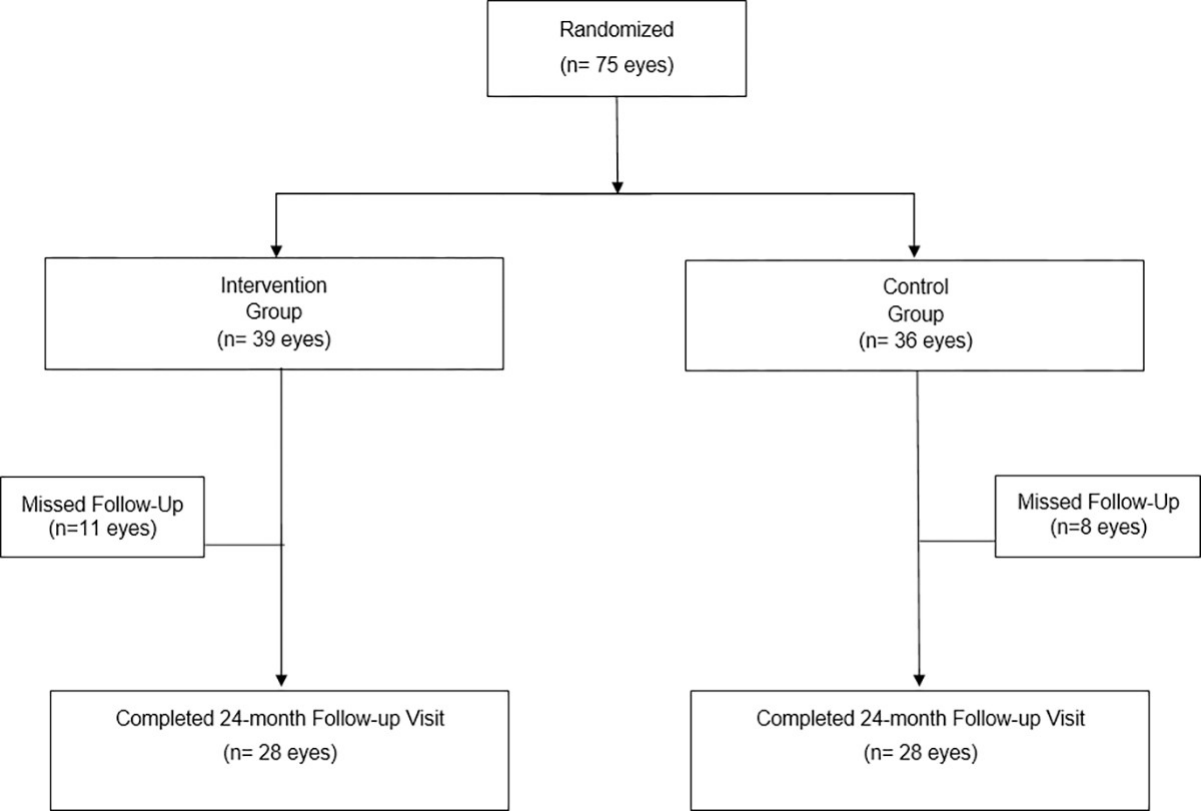
Flowchart of the participants’ progress in the study.

A total of 75 eyes of 63 different patients were included between January 2017 and February 2018. After randomization according to the originally assigned groups, 39 eyes were allocated to the intervention group and 36 eyes to the control group. Twelve patients had both eyes eligible for surgery. When these patients were considered, 4 patients had both eyes included in the intervention group, 2 patients had both eyes included in the control group and 6 patients had one eye allocated in each group. Data regarding demographic and ocular parameters are presented at Table 1. The number of patients was considered for demographic parameters and the number of eyes included in the study for the ocular parameters. There was no statistically significant difference in clinical and demographic characteristics between groups at baseline (Table 1). The flowchart of patients’ progress is depicted in Fig 1.

**Table 1:** Baseline demographic and ocular data of study patients.

	**Intervention Group (n = 35 patients)**	**Control Group (n = 34 patients)**	**P value**
Age (years)	63.2 ± 9.82	67.4 ± 11.5	0.76
Gender (%; M/F)	51/49	56/44	0.81
Race (%; White / Black / Others)	46/25/29	44/28/28	0.97
Diagnosis (%; POAG / PACG / Others)	49/31/20	71/23/6	0.11
	**Intervention Group (n = 39 eyes)**	**Control Group (n = 36 eyes)**	**P value**
Baseline IOP (mmHg)	21.3 ± 7.2	20.4 ± 6.4	0.46
MD index (dB)	-13.9 ± 8.0	-15.2 ± 8.4	0.49
VFI index (%)	58.0 ± 27.4	54.3 ± 27.5	0.43
Central corneal thickness (μm)	501.4 ± 29.9	510.4 ± 29.6	0.34

M, male; F, female; POAG, primary open-angle glaucoma; PACG, primary angle-closure glaucoma; IOP, intraocular pressure; MD, mean deviation; VFI, visual field index.

Data given as mean ± standard deviation whenever indicated.

At the 24-month follow-up, mean IOP was 10.5 ± 3.7 mmHg in the intervention group and 9.3 ± 3.7 mmHg in the control group, while number of hypotensive medications was 0.10 ± 0.31 in the intervention group and 0.64 ± 1.2 in the control group. Data regarding IOP and number of medications at all timepoints are presented at Table 2.

**Table 2:** Intraocular pressure and number of medications data at all timepoints.

	Intervention Group (n = 39)	Control Group (n = 36)
***IOP***		
Baseline	21.3 ± 7.2	20.4 ± 6.4
6 months	9.9 ± 4.8	10.8 ± 4.5
12 months	11.1 ± 5.1	11.4 ± 4.6
18 months	10.6 ± 3.5	9.5 ± 3.8
24 months	10.5 ± 3.7	9.3 ± 3.7
***Number of medications***		
Baseline	3.2 ± 0.5	3.1 ± 0.8
6 months	0.1 ± 0.5	0.3 ± 0.6
12 months	0.3 ± 0.8	0.5 ± 0.9
18 months	0.1 ± 0.2	0.6 ± 1.0
24 months	0.1 ± 0.3	0.6 ± 1.2

IOP, intraocular pressure.

Multivariable regression analysis demonstrated a significant association between follow-up IOP and type of intervention when the number of medications was evaluated as a covariate at 18 (P < 0.001, Table 3) and 24 months (P < 0.001, Table 4). This analysis indicates that IOP levels were significantly lower in the intervention group when compared to the control group at 18 and 24 months of follow-up when number of medications was considered as a covariate.

**Table 3:** Results of multivariable random coefficient model analysis of type of intervention, baseline IOP and 18-month follow-up IOP, including the covariable of number of medications.

Type of Intervention	Coefficient	95% CI	P value
**Baseline IOP**	-0.006	-0.03–0.01	0.494
**18-month follow-up IOP**	-0.048	-0.09 –-0.01	0.017
**Number of medications**	0.313	0.15–0.48	<0.001
**Constant**	1.010	0.57–1.44	<0.001

IOP, intraocular pressure; CI, confidence interval.

**Table 4:** Results of multivariable random coefficient model analysis of type of intervention, baseline IOP and 24-month follow-up IOP, including the covariable of number of medications.

Type of Intervention	Coefficient	95% CI	P value
**Baseline IOP**	-0.011	-0.03–0.01	0.180
**24-month follow-up IOP**	-0.039	-0.07 –-0.01	0.021
**Number of medications**	0.237	0.09–0.37	0.001
**Constant**	1.037	0.06–1.50	<0.001

IOP, intraocular pressure; CI, confidence interval.

**Fig 2:**
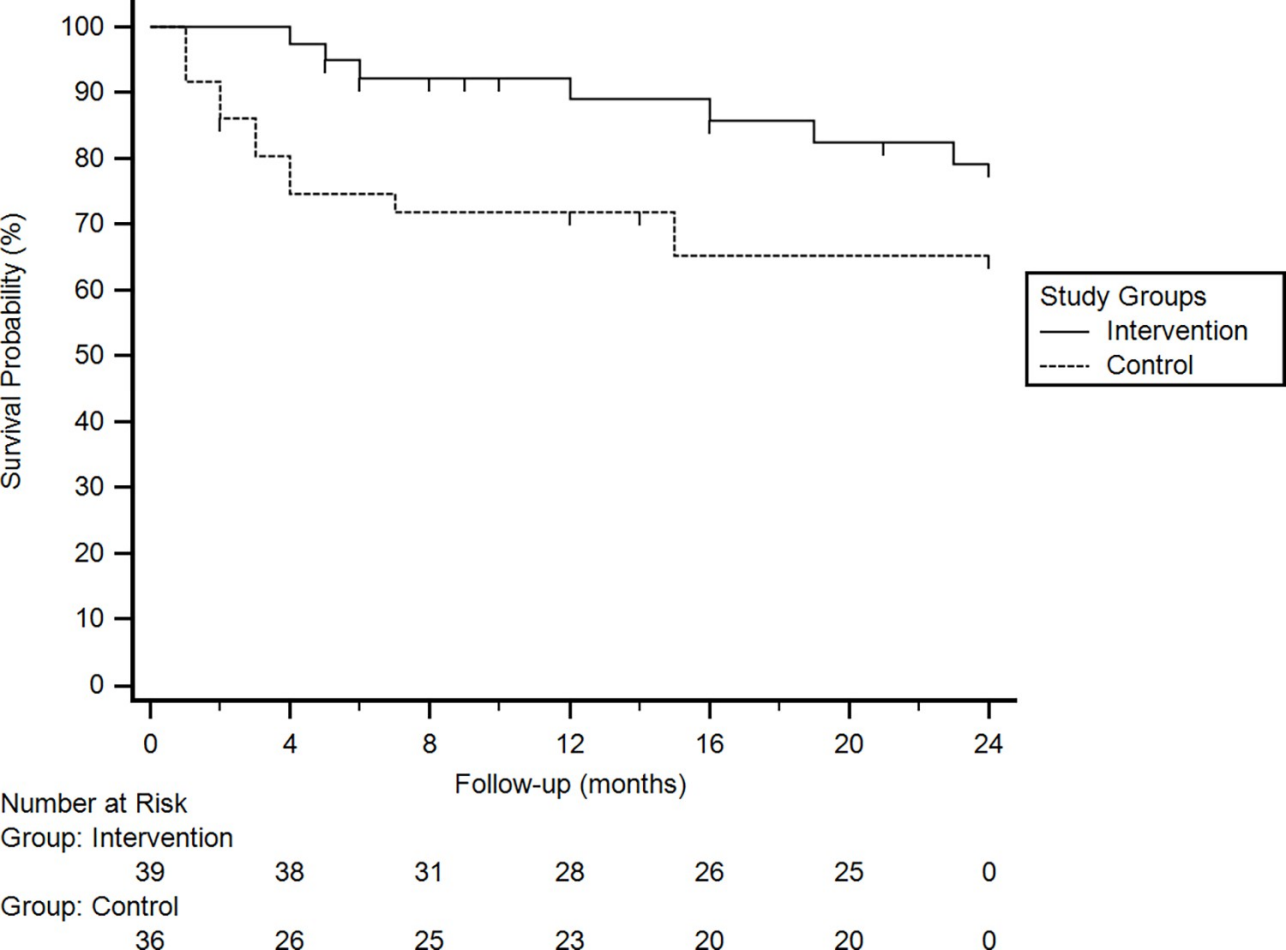
Kaplan-Meier plot of the cumulative probability of complete surgical success at the 24-month follow-up.

Comparing the postoperative success rates between groups, higher complete success rates were found in the intervention group. This difference was statistically significant (cox regression analysis) at 06 (90.9% vs 68.7%; p = 0.03), 12 (87.2% vs 66.7%; p = 0.02) and 18 months (87.2% vs 66.7%; p = 0.02). Although success rates at 24 months were higher in the intervention group (82.0% vs 66.7%; p = 0.09), this difference did not reach statistical significance. When qualified success criteria were considered, no difference was found in success rates between groups (Table 5). The Kaplan-Meier survival plot for complete success rates at the 24-month follow-up is presented in Fig 2.

**Table 5:** Comparison of surgical success rates between groups at all timepoints.

	06 months			12 months			18 months			24 months		
	Intervention Group	Control Group	p	Intervention Group	Control Group	p	Intervention Group	Control Group	p	Intervention Group	Control Group	p
Complete	90.91	68.75	*0*.*03*	87.18	66.67	*0*.*02*	87.18	66.67	*0*.*02*	82.05	66.67	0.09
Qualified	93.94	87.50	0.35	89.74	83.33	0.43	89.74	83.33	0.47	84.62	80.56	0.70

IOP, intraocular pressure

Success rates shown as percentiles (%) and p values based on cox regression analysis

An analysis of the eyes that underwent surgery with IOP ≤ 15 mmHg due to end-stage glaucoma and/or progression was also performed. A total of 13 eyes were included in this condition (7 in the intervention group and 6 in the control group). Considering the eyes in the intervention group, mean IOP was 12.8 ± 1.7 at baseline and 10.1 ± 2.9 mmHg at the 24-month follow-up. Additionally, mean number of medications was 3.3 ± 0.7 at baseline and no patient was on medication, at the 24-month follow up. Therefore, complete success rates were 100% at the 24-month follow-up. Conversely, while considering the eyes in the control group, mean IOP was 13.6 ± 1.9 at baseline and 8.1 ± 4.9 mmHg at the 24-month follow-up. Additionally, mean number of medications was 3.3 ± 0.5 at baseline and 0.6 ± 1.2 at the 24-month follow up. Two (33.3%) patients filled the qualified success and 4 (66.6%) patients filled the complete success criteria. Therefore, no difference was found between groups regarding IOP and number of medication in this analysis (p>0.17) and higher complete success rates were found in the intervention group (p = 0.09), similar to the results found in the overall sample.

Regarding needling and reoperation rates due to uncontrolled IOP, there was no difference between the intervention and control groups (15.4% vs 13.9%—p = 1.00; 10.2% vs 11.1%—p = 1.00; respectively). Of note, the need for suture removal in the control group was higher than in the intervention group (41.0% vs 63.9%; p = 0.06) and there was no significant difference regarding the duration of postoperative topical corticosteroid use between the intervention and control groups (63.9 ± 12.9 vs 67.0 ± 13.8 days–p = 0.70; respectively). When overall postoperative complications were analyzed, there was also no difference between both groups (46.1% vs 41.7%; p = 0.81) and no difference was found when specific complication rates and sight threatening events were analyzed either (Table 6).

**Table 6:** Comparison of complication rates between groups.

	Intervention Group (n = 39)	Control Group (n = 36)	P value
***Postoperative complications***			
Hyphema	2 (5.1%)	4 (11.1%)	0.42
Persistent Bleb leakage	1 (2.5%)	0 (0.0%)	0.97
Corneal *dellen*	2 (5.1%)	2 (5.5%)	1.00
***Sight threatening adverse events***			
Misdirection glaucoma	0 (0%)	1 (2.8%)	0.97
Hypotonic maculopathy	5 (13.9%)	3 (8.3%)	0.71
Choroidal detachment	1 (2.5%)	1 (2.8%)	0.51
Endophthalmitis	0 (0%)	0 (0%)	-
Loss of light perception	0 (0%)	0 (0%)	-

During the 24-month follow-up period, 11 eyes in the intervention group and 8 eyes in the control group lost follow-up and did not complete the 2-year follow-up visit. Regarding these eyes, in the intervention group, follow-up duration varied from 5 to 21 months and, at the last follow-up visit, all eyes had IOP between 6 and 14 mmHg without the need for medication, except for one eye which had IOP of 22 mmHg with 3 medications. On the other hand, in the control group, follow-up duration varied from 2 to 17 months and, at the last follow-up visit, 4 eyes had IOP between 5 to 11 without medication, while 4 eyes had IOP ranging from 8 to 19 with 2 medications. Finally, it must be highlighted that most of these cases that lost follow-up in both groups could be explained by the fact that a significant proportion of study patients were referred from cities other than that where the study Eye Center is located.

## Discussion

Modulation of wound healing is paramount for surgical success in glaucoma surgery. Therefore, subtenon cicatrization in the postoperative period of TRAB surgery is a frequent concern for the glaucoma specialist. It is well-known that, although surgeries are usually performed using a same standardized technique, results may vary significantly. In order to address this issue, antifibrotic and anti-inflammatory agents are widely employed, such as MMC, 5-fluoracil and corticosteroids. In this randomized clinical trial with non-inflammatory glaucoma patients, we found that the adjuvant use of subtenon TAAC injection at the bleb site at the end of surgery significantly improved surgical outcomes when compared to isolated MMC.

The use of subtenon TAAC in TRABs has been evaluated in a few previous studies. Of note, surgical technique, study design, follow-up time and patients profile varied significantly between these reports. Initially, the isolated use of subtenon TAAC in TRABs without MMC was evaluated by Giangiacomo and colleagues, and the authors reported about 93% success rates after 6 months [26]. Additionally, when retrospectively comparing the isolated use of MMC to the isolated use of subtenon TAAC, Hogewind and colleagues [8] reported similar success rates between both techniques after a 5-year follow-up period, suggesting that subtenon TAAC may be at least as effective as MMC in lowering IOP in primary glaucomas. The overall results of these studies were encouraging for further investigation of the adjuvant use of intraoperative TAAC in primary MMC-TRABs.

In this context, a few clinical studies have evaluated specifically the adjuvant effect of subtenon TAAC in MMC-TRABs. Initially, Tham and colleagues [27] reported an IOP reduction of approximately 50% at 3 months of follow-up, in a case-series of 11 patients that had undergone TRABs and other glaucoma procedures. Additionally, success rates from 80% to 93% were reported in retrospective case-series in high-risk eyes, after 12 and 6 months of follow-up, respectively [28, 29]. Finally, Yuki and colleagues [30] conducted a randomized clinical trial and found similar success rates between MMC-TRABs with and without TAAC after a 12-month follow-up period in eyes with secondary glaucomas. All these information considered, it is reasonable to state that, although most of the available data comes from low-quality evidence (small case-series and retrospective studies), it had revealed that subtenon TAAC could be potentially used to enhance TRABs success rates. The only randomized clinical trial assessing this technique enrolled a higher risk of failure population (patients with secondary glaucomas), which might explain the difference in our success rates.

Based on the 24-month data observed in this randomized controlled trial, we believe our data provides evidence supporting the utilization of subtenon TAAC as an adjuvant in MMC-TRABs in non-inflammatory glaucomas. First, the multivariate analysis demonstrated significantly lower IOP levels in the intervention group when compared to the control group at 18 and 24 months of follow-up when number of medications was considered as a covariate. Second, success rates were significantly higher in the intervention group when compared to the control group. Of note, our sample size was calculated based on IOP difference between the 2 groups, and the sample size necessary to show a significant difference in success rates between groups might have been larger. Nonetheless, even in this context of small sample size, we were able to demonstrate a significant difference in success rates between groups at 6, 12 and 18 months. Hence, it might be considered that the borderline significance level at 24 months could be because of small sample size. Additionally, analyzing the cases of failure of complete success, we found that they occurred at relatively lower pressure levels, and therefore, many patients could achieve qualified success when using antiglaucoma medications.

Regarding the rates of adverse events, we found that the adjuvant injection of subtenon TAAC did not affect TRABs safety profile. There was no difference in intraoperative complications and most postoperative complications were solved clinically, with no difference between groups. One patient in the control group developed misdirection glaucoma, which was reversed with anterior hyaloidectomy through YAG laser. All the other cases requiring surgical intervention were cases of hypotonic maculopathy. In this context, 5 (13.9%) patients in the intervention group and 3 (8.3%) patients in the control group had the maculopathy reversed with restrictive transconjunctival sutures, associated with choroidal drainage whenever necessary. Even though hypotony rates did not differ between groups, one important aspect that must be highlightened is that, when a patient presents with overfiltration, the usually employed initial treatment of reducing the corticosteroids drops administration to stimulate subtenon fibrosis may have a limited effect due to the subtenon TAAC deposit. Following the same rationale of continuous corticosteroid release, we must highlight that we do not recommend the use of intraoperative subtenon TAAC to isolated needling (even though this was not evaluated in our study), as these cases have a higher risk of early failure and would be prone to corticosteroid-induced IOP spikes.

In the face of our results, we believe it is important to discuss the main implications of our findings. First, since our results have shown that success rates were improved by the adjuvant use of TAAC, one might consider adopting this procedure as a standard technique for primary TRABs in patients with non-inflammatory glaucomas. Additionally, we believe that our findings might significantly impact the postoperative regimen of corticosteroid eyedrops in these patients. Currently, patients apply topical corticosteroids at every 1 or 2 hours at the initial postoperative period of TRAB surgery. Considering the fact that TAAC is a deposit formulation which persists in the subtenon space for a few months, its intraoperative use could mitigate the need for corticosteroid eyedrops postoperatively. Notably, this hypothesis was not evaluated in our study and further investigation is necessary in order to clarify this issue.

The present study has some specific limitations that should be addressed. First, we did not perform a masked evaluation of the patients neither evaluated the morphologic characteristics of the filtering bleb. These limitations derived from the same factor: subtenon TAAC forms a whitish easily visible deposit. In that context, it would be impossible to perform clinical evaluation and bleb morphology classification without noticing the presence of TAAC in the subtenon space. Hence, the bleb morphology classification would possibly lead to a biased evaluation, we chose not to perform it. Second, we did not perform a subtenon placebo injection of balanced saline solution at the end the surgery for the control group. We chose not to perform such sham procedure due to its potential unnecessary adverse effects, such as intrableb bleeding and bleb leakage. Third, randomization was performed by flip-coin technique. Although the technique is effective in avoiding selection bias, it might not be ideal since it might lead to an asymmetric distribution of patients between groups. Nonetheless, since our samples were symmetric (39 eyes in the intervention group and 36 eyes in the control group), we believe it did not have any impact in our results. Fourth, we did not define a cut-off IOP as an inclusion criterion. Since TRABs are indicated not only in eyes with high IOPs but also in eyes with anatomical or functional progression, a real life scenario with better external validation would include these eyes with lower IOPs but still presenting progression. In order to mitigate this issue, instead of choosing usual IOP-based criteria such as 21 or 18 mmHg, we opted for a more strict success criterion of IOP ≤ 15 mmHg and performed an analysis of the results of this specific group of patients, which revealed the same pattern of the general sample. Therefore, we believe this issue did not impact our final results. Finally, the fact that the study surgical procedures were performed by multiple surgeons should be considered while interpreting our findings, as it might have influenced surgical outcomes. However, we believe that the fact that the same experienced surgeon was present in all procedures and all surgeries followed a standardized protocol likely mitigated this issue.

In conclusion, this study found significantly lower IOPs levels at 18 and 24 months of follow-up and higher complete success rates until 18 months of follow-up with the use of subtenon TAAC as an adjuvant to standard MMC-TRABs in non-inflammatory glaucoma patients. Mainly, our results may help clinicians to achieve better outcomes in TRAB surgery. Additionally, we provide evidence for future studies to evaluate whether subtenon TAAC injection may allow a less strict postoperative regimen of corticosteroid eyedrops following TRAB surgery.

## Supporting information

S1 File CONSORT checklist. (DOC)

S2 File Study protocol. This file presents the protocol submitted for approval to the ethics committee.
(DOC)

S3 File Study protocol in english. This file presents the main points of protocol submitted for approval to the ethics committee.
(DOC)

S4 File Study dataset. This file presents the study’s minimal underlying dataset.
(XLS)

S5 File Study data by surgeon. This file presents surgical success data of each surgeon in the study.
(DOC)

## References

[1] Kingman S (2004) Glaucoma is second leading cause of blindness globally. Bull World Health Organ 82: 887–888. doi: /S0042-96862004001100019 15640929 PMC2623060

[2] Tham YC, Li X, Wong TY, Quigley HA, Aung T, et al. (2014) Global prevalence of glaucoma and projections of glaucoma burden through 2040: a systematic review and meta-analysis. Ophthalmology 121: 2081–2090. doi: 10.1016/j.ophtha.2014.05.013 24974815

[3] Feiner L, Piltz-Seymour JR, Collaborative Initial Glaucoma Treatment S (2003) Collaborative Initial Glaucoma Treatment Study: a summary of results to date. Curr Opin Ophthalmol 14: 106–111. doi: 10.1097/00055735-200304000-00010 12698052

[4] Lichter PR, Musch DC, Gillespie BW, Guire KE, Janz NK, et al. (2001) Interim clinical outcomes in the Collaborative Initial Glaucoma Treatment Study comparing initial treatment randomized to medications or surgery. Ophthalmology 108: 1943–1953. doi: 10.1016/s0161-6420(01)00873-9 11713061

[5] Schwartz K, Budenz D (2004) Current management of glaucoma. Curr Opin Ophthalmol 15: 119–126. doi: 10.1097/00055735-200404000-00011 15021223

[6] Hosseini H, Mehryar M, Farvardin M (2007) Focus on triamcinolone acetonide as an adjunct to glaucoma filtration surgery. Med Hypotheses 68: 401–403. doi: 10.1016/j.mehy.2006.04.075 16919396

[7] Lama PJ, Fechtner RD (2003) Antifibrotics and wound healing in glaucoma surgery. Surv Ophthalmol 48: 314–346. doi: 10.1016/s0039-6257(03)00038-9 12745005

[8] Hogewind BF, Pijl B, Hoyng CB, Theelen T (2013) Purified triamcinolone acetonide as antifibrotic adjunct in glaucoma filtering surgery. Graefes Arch Clin Exp Ophthalmol 251: 1213–1218. doi: 10.1007/s00417-012-2161-y 23052714

[9] Jones E, Clarke J, Khaw PT (2005) Recent advances in trabeculectomy technique. Curr Opin Ophthalmol 16: 107–113. doi: 10.1097/01.icu.0000156138.05323.6f 15744141

[10] Cheung JC, Wright MM, Murali S, Pederson JE (1997) Intermediate-term outcome of variable dose mitomycin C filtering surgery. Ophthalmology 104: 143–149. doi: 10.1016/s0161-6420(97)30347-9 9022119

[11] Matsuda T, Tanihara H, Hangai M, Chihara E, Honda Y (1996) Surgical results and complications of trabeculectomy with intraoperative application of mitomycin C. Jpn J Ophthalmol 40: 526–532. 9130057

[12] Perkins TW, Gangnon R, Ladd W, Kaufman PL, Heatley GA (1998) Trabeculectomy with mitomycin C: intermediate-term results. J Glaucoma 7: 230–236. 9713779

[13] Araujo SV, Spaeth GL, Roth SM, Starita RJ (1995) A ten-year follow-up on a prospective, randomized trial of postoperative corticosteroids after trabeculectomy. Ophthalmology 102: 1753–1759. doi: 10.1016/s0161-6420(95)30797-x 9098274

[14] Roth SM, Spaeth GL, Starita RJ, Birbillis EM, Steinmann WC (1991) The effects of postoperative corticosteroids on trabeculectomy and the clinical course of glaucoma: five-year follow-up study. Ophthalmic Surg 22: 724–729. 1787937

[15] Starita RJ, Fellman RL, Spaeth GL, Poryzees EM, Greenidge KC, et al. (1985) Short- and long-term effects of postoperative corticosteroids on trabeculectomy. Ophthalmology 92: 938–946. doi: 10.1016/s0161-6420(85)33931-3 4022581

[16] Ferrante P, Ramsey A, Bunce C, Lightman S (2004) Clinical trial to compare efficacy and side-effects of injection of posterior sub-Tenon triamcinolone versus orbital floor methylprednisolone in the management of posterior uveitis. Clin Exp Ophthalmol 32: 563–568. doi: 10.1111/j.1442-9071.2004.00902.x 15575824

[17] Bonini-Filho MA, Jorge R, Barbosa JC, Calucci D, Cardillo JA, et al. (2005) Intravitreal injection versus sub-Tenon’s infusion of triamcinolone acetonide for refractory diabetic macular edema: a randomized clinical trial. Invest Ophthalmol Vis Sci 46: 3845–3849. doi: 10.1167/iovs.05-0297 16186372

[18] Asano S, Miyake K, Miyake S, Ota I (2007) Relationship between blood-aqueous barrier disruption and ischemic macular edema in patients with branch or central retinal vein occlusion: effects of sub-tenon triamcinolone acetonide injection. J Ocul Pharmacol Ther 23: 577–584. doi: 10.1089/jop.2007.0057 18001246

[19] Foster PJ, Buhrmann R, Quigley HA, Johnson GJ (2002) The definition and classification of glaucoma in prevalence surveys. Br J Ophthalmol 86: 238–242. doi: 10.1136/bjo.86.2.238 11815354 PMC1771026

[20] Swanson MW (2011) The 97.5th and 99.5th percentile of vertical cup disc ratio in the United States. Optom Vis Sci 88: 86–92. doi: 10.1097/OPX.0b013e3181fc3638 20966802

[21] (2017) European Glaucoma Society Terminology and Guidelines for Glaucoma, 4th Edition—Chapter 3: Treatment principles and options Supported by the EGS Foundation: Part 1: Foreword; Introduction; Glossary; Chapter 3 Treatment principles and options. Br J Ophthalmol 101: 130–195. doi: 10.1136/bjophthalmol-2016-EGSguideline.003 28559477 PMC5583689

[22] Fea AM (2010) Phacoemulsification versus phacoemulsification with micro-bypass stent implantation in primary open-angle glaucoma: randomized double-masked clinical trial. J Cataract Refract Surg 36: 407–412. doi: 10.1016/j.jcrs.2009.10.031 20202537

[23] Association. WG (2009.) Guidelines on Design and Reporting of Glaucoma Surgical Trials. In: Shaarawy TM, Sherwood MB, Grehn F,. eds Amsterdam: Kugler Publications;.

[24] Wang Q, Harasymowycz P (2016) Collagen Cross-linking for Late-onset Bleb Leakage: 1-Year Results. J Glaucoma 25: e273–276. doi: 10.1097/IJG.0000000000000295 26066500

[25] Field CA WA (2007) Bootstrapping clustered data. Journal of the Royal Statistical Society: Series B (Statistical Methodology) 69(3): 369–390.

[26] Giangiacomo J, Dueker DK, Adelstein E (1986) The effect of preoperative subconjunctival triamcinolone administration on glaucoma filtration. I. Trabeculectomy following subconjunctival triamcinolone. Arch Ophthalmol 104: 838–841. doi: 10.1001/archopht.1986.01050180072032 3718307

[27] Tham CC, Li FC, Leung DY, Kwong YY, Yick DW, et al. (2006) Intrableb triamcinolone acetonide injection after bleb-forming filtration surgery (trabeculectomy, phacotrabeculectomy, and trabeculectomy revision by needling): a pilot study. Eye (Lond) 20: 1484–1486. 16691258 10.1038/sj.eye.6702372

[28] Kahook MY, Camejo L, Noecker RJ (2009) Trabeculectomy with intraoperative retrobulbar triamcinolone acetonide. Clin Ophthalmol 3: 29–31. 19668541 PMC2709002

[29] Keorochana N, Kunasuntiwarakul S, Treesit I, Choontanom R (2017) The efficacy of preoperative posterior subtenon injection of triamcinolone acetonide in noninfectious uveitic patients with secondary glaucoma undergoing trabeculectomy. Clin Ophthalmol 11: 2057–2063. doi: 10.2147/OPTH.S145957 29200819 PMC5701565

[30] Yuki K, Shiba D, Kimura I, Ohtake Y, Tsubota K (2009) Trabeculectomy with or without intraoperative sub-tenon injection of triamcinolone acetonide in treating secondary glaucoma. Am J Ophthalmol 147: 1055–1060, 1060 e1051-1052 doi: 10.1016/j.ajo.2009.01.007 19327739

