# Supplementary material for: Subtenon triamcinolone as an adjuvant in mitomycin-C-enhanced trabeculectomy in non-inflammatory glaucomas: A randomized clinical trial
Source: PLoS One. 2022 May 26;17(5):e0268623. doi: 10.1371/journal.pone.0268623 (PMC9135266; doi:10.1371/journal.pone.0268623)
Supplement: S2 File — This file presents the protocol submitted for approval to the ethics committee. (DOC) [file pone.0268623.s002.doc]

**TITULO DO PROJETO DE PESQUISA:**

USO DA Triancinolona Subtenoniana COMO ADJUVANTE NO PROCEDIMENTO DE Trabeculectomia EM PACIENTES COM GlaucomaS PRIMÁRIOS: Um ensaio clinico randomizado

**EQUIPE DE PESQUISA:**

- Aluno candidato ao Doutorado Acadêmico do Departamento de Oftalmologia da UNIFESP/EPM: Dr Diego Torres Dias

- Orientador do projeto de pesquisa: Prof Dr Tiago Prata

- Demais membros da equipe de pesquisa: Dra Michele Ushida, Dra Izabela Almeida, Dr Flávio Siqueira Lopes, Dr Fábio Kanadani

**INSTITUIÇÃO:**

Setor de Glaucoma - Departamento de Oftalmologia - UNIFESP/EPM

**LOCAL E DATA:**

São Paulo – SP; Setembro de 2017

**RESUMO**

**OBJETIVO**

Avaliar o efeito do uso adjuvante da triancinolona subtenoniana no sucesso cirúrgico de pacientes com glaucomas primários submetidos a trabeculectomia.

**MÉTODO**

Ensaio Clínico Unicêntrico Randomizado. Serão recrutados pacientes consecutivos com diagnóstico de glaucomas primários e com indicação de cirurgia de trabeculectomia entre outubro de 2017 e fevereiro de 2018. Os pacientes serão distribuídos entre 2 grupos de modo randomizado, podendo ser alocados para realização de cirurgia de trabeculectomia com mitomicina ou para realização de trabeculectomia com mitomicina mais triancinolona subtenoniana, ao final da cirurgia. A pressão intraocular pré-operatória e com 1, 3, 6, 12, 18 e 24 meses após a cirurgia e a quantidade de medicação antiglaucomatosa utilizada nesses 6 momentos pelos dois grupos serão avaliadas. Será realizada análise computadorizada (*software* MedCalc; MedCalc Inc., Mariakerke, Bélgica), sendo adotado *P*<0,05 como nível de significância estatística.

SUMÁRIO

| 1. Introdução | ........... | 04 |
| --- | --- | --- |
| - 1. Objetivo   2. Justificativa | ...........  ........... | 05 05 |
| 1. Casuística e método | ........... | 05 |
| - 1. Sujeitos      1. Observância de normas éticas e regulamentares      2. Casuística e amostragem      3. Seleção de sujeitos   2. Procedimentos      1. Avaliação oftalmológica: procedimentos básicos         1. Medida da acuidade visual         2. Biomicroscopia do segmento anterior         3. Tonometria de aplanação         4. Gonioscopia         5. Fundo de olho         6. Campo visual         7. Retinografia         8. Paquimetria      2. Intervenção cirúrgica         1. Trabeculectomia   3. Desfechos e definições de sucesso e falência   4. Análise dos dados | ...........  ...........  ...........  ...........  ...........  ...........  ...........  ...........  ...........  ...........  ...........  ...........  ...........  ...........  ...........  ...........  ...........  ........... | 05  05  06  06  08  08  08  08  09  09  10  10  10  10  11  11  12  13 |
| 1. Cronograma | ........... | 15 |
| 1. Orçamento | ........... | 16 |
| 1. Referências bibliográficas | ........... | 17 |

1. INTRODUÇÃO

O glaucoma é a maior causa de cegueira irreversível no mundo.[^1^](#_ENREF_1) Estima-se que cerca de 64.3 milhões de pessoas entre 40 e 80 anos tenham glaucoma e que esse número irá aumentar para 76 milhões, em 2020, e 111.8 milhões, em 2040.[^2^](#_ENREF_2)

As opções terapêuticas para estes pacientes incluem o tratamento clínico e o tratamento cirúrgico, que pode ser feito a laser e com cirurgias incisionais. De um modo geral, o tratamento inicial é clinico, estando as cirurgias incisionais indicadas para os casos em que não foi alcançado um controle satisfatório da doença com o tratamento clínico e a laser. No que se refere às opções de cirurgia incisional, há mais de 20 anos, a trabeculectomia é a técnica mais utilizada mundialmente.[^5^](#_ENREF_5)

A trabeculectomia tem uma peculiaridade que a diferencia de grande parte das cirurgias: seu sucesso depende da inibição controlada do processo cicatricial. Isto acontece porque este procedimento visa criar uma fístula protegida que funciona como um nova via de drenagem para o humor aquoso a fim de reduzir a pressão intraocular (PIO) destes pacientes.[^8^](#_ENREF_8) Deste modo, a falência da cirurgia está comumente relacionada à cicatrização do espaço subtenoniano, impedindo um fluxo adequado por esta fístula.[^9^](#_ENREF_9) Por conta disso, o uso de medicações antifibróticas intraoperatórias (a mais amplamente utilizada é a mitomicina C)[^10-12^](#_ENREF_10) e de corticoides tópicos pós operatórios[^13-15^](#_ENREF_13) fazem parte do dia a dia do cirurgião de glaucoma, na medida em que aumentam as taxas de sucesso da trabeculectomia. Deve se ter em mente, entretanto, que a introdução dos antimetabólitos também está associada a um aumento de complicações cirúrgicas,[^16^](#_ENREF_16) e que mesmo com estas medidas, muitas cirurgias falham por conta de cicatrização excessiva.[^7^](#_ENREF_7)

A injeção subtenoniana de acetato de triancinolona além de ser uma opção terapêutica segura e eficaz já bem estabelecida no tratamento de diversas condições oftalmológicas (por exemplo, uveítes posteriores,[^17^](#_ENREF_17) edema macular diabético[^18^](#_ENREF_18) e edema macular após oclusões venosas[^19^](#_ENREF_19)), aparece como uma possível medida capaz de auxiliar no sucesso das cirurgias de glaucoma. Alguns poucos estudos já avaliaram seu uso em pacientes submetidos a trabeculectomia, com resultados inconsistentes.[^20-22^](#_ENREF_20) Acreditamos que esses dados conflitantes possam advir do fato de que a maior parte dessas publicações não investigou a triancinolona como adjuvante a mitomicina durante a trabeculectomia, mas sim a comparou diretamente com a mitomicina. Um único ensaio clínico randomizado foi publicado avaliando esta técnica,[^23^](#_ENREF_23) porém neste estudo foram incluídos apenas pacientes com glaucoma secundário, por ser um grupo de mais difícil controle.

- 1. OBJETIVO

Objetiva-se com o presente trabalho avaliar o efeito do uso adjuvante da triancinolona subtenoniana no sucesso cirúrgico de pacientes com glaucomas primários submetidos a trabeculectomia com mitomicina.

- 1. JUSTIFICATIVA

O acetato de triancionolona tem sido usado com muita segurança e eficácia no tratamento de outras patologias oftalmológicas. Seu uso no intraoperatório de cirurgias antiglaucomatosas se apresenta como uma técnica que pode contribuir para o sucesso das trabeculectomias, como observado em avaliação retrospectiva prévia realizada pelo nosso grupo. Nesse contexto, se fazem necessários estudos prospectivos bem controlados para avaliar o efeito do uso adjuvante do acetato de triancinolona subtenoniano intraoperatório nas taxas de sucesso de cirurgias de trabeculectomia com mitomicina em pacientes com glaucoma primário.

1. CAUSUÍSTICA E MÉTODOS
   1. SUJEITOS
      1. OBSERVÂNCIA DE NORMAS ÉTICAS E REGULAMENTARES

Todos os pacientes participantes da presente pesquisa serão estudados segundo os preceitos da Declaração de Helsinque (1964), observando-se também as Normas de Pesquisa Envolvendo Seres Humanos estabelecidas na Resolução 466/12 do Conselho Nacional de Saúde. O projeto, submetido à aprovação do “Comitê de Ética em Pesquisa” da Universidade Federal de São Paulo, será do conhecimento dos pacientes (ou seus responsáveis habilitados), por meio de termo de consentimento livre e esclarecido (TCLE), cujo teor caberá ao pesquisador esclarecer.

- - 1. CASUÍSTICA E AMOSTRAGEM

Será realizado um ensaio clínico unicêntrico randomizado. Serão recrutados pacientes consecutivos acompanhados no setor de glaucoma do Hospital Medicina dos Olhos de Osasco (Osasco, São Paulo, Brasil) com diagnóstico de glaucomas primários e com indicação de cirurgia de trabeculectomia entre 3 outubro de 2017 e 6 fevereiro de 2018. Os pacientes serão distribuídos entre 2 grupos de modo randomizado, através de sorteio com moeda, podendo ser alocados para realização de cirurgia de trabeculectomia com mitomicina ou para realização de trabeculectomia com mitomicina mais triancinolona subtenoniana, ao final da cirurgia. Parâmetros clínicos e oculares serão documentados no pré-operatório e com 1, 3, 6, 12, 18 e 24 meses. Para o cálculo amostral, a varíável escolhida foi a magnitude de redução pressórica com 2 anos de cirurgia. Considerando um valor de alfa (erro tipo I) de 0,05 e uma diferença média mínima esperada entre os grupos de 3mmHg para um desvio padrão esperado de 4mmHg, serão necessário 29 pacientes em cada grupo para termos um poder da amostra de 80%.

- - 1. SELEÇÃO DE SUJEITOS
       1. Critérios de inclusão
- Ambos os sexos;
- Diagnóstico oftalmológico de glaucoma primário de ângulo aberto (GPAA) ou glaucoma primário de ângulo fechado (GPAF) não controlado clinicamente com indicação de cirurgia de trabeculectomia.
  - Glaucoma será definido pela presença de sinais característicos de neuropatia óptica glaucomatosa (NOG) associado a defeito de campo visual (CV) característico na perimetria computadorizada. A presença de NOG é caracterizada pela presença de relação escavação/disco vertical ≥ 0,6, assimetria entre os olhos ≥ 0,2, defeitos localizados na camada de fibras nervosas peripapilar e/ou defeitos na rima neural do disco óptico, na ausência de outras anormalidades que possam explicar tais achados. Defeitos carateristicos de CV serão definidos como *glaucoma hemifield test* fora dos limites de normalidade e a presença de ao menos 3 pontos contíguos no mesmo hemicampo no gráfico do *pattern deviation* no gráfico *pattern deviation* com p<1%, sendo ao menos 1 destes com p<0,5%, excluindo pontos na margem do campo e aqueles diretamente acima ou abaixo da mancha cega.
  - A ausência de controle clínico é definida pela progressão anatômica através de retinografia, progressão funcional através da perimetria computadorizada ou PIO acima do alvo estabelecido pelo clínico como seguro para o nível de dano apresentado pelo paciente.
    - 1. Critérios de exclusão
- Pacientes portadores de glaucomas secundários;
- Pacientes com GPAA ou GPAF associado a catarata, não controlados clinicamente, e com indicação de cirurgia combinada de catarata e glaucoma;
- Cirurgias intraoculares prévias, exceto facectomia não complicada. Laser e facectomia não complicada não foram consideradas critério de exclusão desde que realizadas a mais de 6 meses.
  1. PROCEDIMENTOS
     1. AVALIAÇÃO OFTALMOLÓGICA

Os pacientes serão submetidos a exame oftalmológico completo, constituido por: (1) medida de acuidade visual com a melhor correção; (2) biomicroscopia com lâmpada de fenda; (3) aferição da PIO pela tonometria de Goldmann; (4) gonioscopia; (5) biomicroscopia de fundo de olho; (6) perimetria automatizada acromática; (7) retinografia; e (8) paquimetria ultrassônica.

- - - 1. MEDIDA DA ACUIDADE VISUAL

A acuidade visual para longe é determinada segundo a tabela de Snellen, cujos optotipos de base literal, em seqüência não-alfabética, são dispostos em ângulos de 1 minuto de arco. O procedimento obedece à graduação de tamanho dos optotipos, iniciando-se com o maior (correspondente à AV de 0,05) até o de menor tamanho (AV de 1), mantendo-se uma distância de 20 pés (ou 6,1m) entre o paciente e o plano de projeção dos optotipos. O registro dos optotipos corretamente reconhecidos representa a melhor acuidade visual morfoscópica do indivíduo examinado. A aferição da acuidade para perto se baseará na sistemática da tabela de Jaeger, sendo anotadas as medidas de acuidade sob a forma dos símbolos J1 a J6 conforme o tamanho da letra que o indivíduo for capaz de distinguir corretamente.

- - - 1. BIOMICROSCOPIA DO SEGMENTO ANTERIOR

Este exame, realizado com o auxílio de lâmpada de fenda, permite a análise de estruturas oculares em diferentes incidências. O equipamento compõe-se de duas partes básicas: (a) um sistema óptico que funciona como microscópio ocular; (b) um potente e diversificado sistema de iluminação que garante a qualidade da microscopia, incluindo filtros de diferentes cores e um braço móvel, vertical, que, ajustado, permite o direcionamento do foco luminoso, de intensidade graduada, para o estudo das regiões anatômicas oculares.

Diferentes parâmetros funcionais e de estruturas ópticas podem ser investigados mediante a regulação da fenda e a diversificação dos feixes luminosos: (a) iluminação direta difusa para estudo da visão geral; (b) iluminação direta focal para visão de detalhes; (c) iluminação indireta difusa por retro-iluminação, que evidencia as estruturas oculares a partir dos reflexos emitidos de áreas mais internas; (d) iluminação indireta difusa focal, que evidencia as bordas e margens das lesões.

- - - 1. TONOMETRIA DE APLANAÇÃO

A medida da pressão intra-ocular se baseia na aplanação através de um cone, sob anestesia tópica à base de cloridrato de proximetacaína, da área central corneana, permitindo um equilíbrio com a força de deslocamento de líquido intra-ocular. A pressão é aferida através do tonômetro de Goldmann acoplado à lâmpada de fenda “Haag-Streit 900”, usando-se corante de fluoresceína. Os resultados são expressos em milímetros de mercúrio, registrando-se os horários de medições.

- - - 1. GONIOSCOPIA

A gonioscopia é uma ferramenta diagnóstica essencial na avaliação de pacientes com glaucoma. Baseia-se no uso de uma lente em contato com a superfície ocular para visualização das estruturas do ângulo da câmara anterior. Desse modo permite classificar o paciente com glaucoma em GPAA, GPAF ou identificar causas secundárias de glaucoma. Esse exame é realizado com o auxílio da lâmpada de fenda, sob anestesia tópica à base de cloridrato de proximetacaína. Deve-se controlar o ambiente, mantendo-se a sala de exames escura, o feixe de luz da lâmpada de fenda em 1x1mm, e ter cuidado para não iluminar a pupila, desse modo evita-se miose e uma falsa abertura do ângulo. Nos casos de ângulo fechado (definido pela não visualização do trabeculado posterior em ao menos 180º do ângulo), procede-se com a gonioscopia de indentação para diferenciar entre fechamento aposicional e sinequial.

- - - 1. FUNDO DE OLHO

A biomicroscopia de fundo é realizada com o recurso da lâmpada de fenda e de lente de não contato marca Volk 78D, para avaliação do disco óptico e camada de fibras nervosas peripapilar.

- - - 1. CAMPO VISUAL

A perimetria automatizada acromática avalia o campo visual enquanto ferramenta contra potenciais doenças que causam cegueira. Os estímulos são pequenos pontos não móveis, que percorrem a ilha de visão ou campo visual. O ponto aumenta em tamanho ou luminância até o paciente ver o primeiro ponto, que indica o limiar para esse ponto no campo visual. Esta estratégia permite uma medida quantitativa da densidade relativa de um defeito.

- - - 1. RETINOGRAFIA

A retinografia corresponde a uma fotografia do fundo do olho. É um método excelente para documentação e acompanhamento da aparência do fundo de olho do paciente ao longo do tempo. É importante pois permite uma avaliação mais detalhada das características anatômicas do disco óptico e região peripapilar, além de, comparando imagens atuais e antigas, poder detectar mudanças sutis no disco óptico. O exame é realizado sob midríase, utilizando-se duas gotas de tropicamida a 1% instiladas a intervalo de cinco minutos em cada olho, utilizando-se o aparelho Visucam Lite (Carl Zeiss Meditec; AG07740, Jena, Germany).

- - - 1. PAQUIMETRIA

A paquimetria é o exame que mede a espessura corneana. É uma medida muito importante na avaliação de pacientes com glaucoma pois influencia na acurácia da medida da tonometria de aplanação e acredita-se que seja um fator de risco independente para desenvolvimento e progressão do glaucoma. Este exame pode ser realizado com tecnologia óptica ou ultrassônica, sendo esta última mais precisa na determinação da espessura corneana.

- - 1. INTERVENÇÃO CIRÚRGICA
       1. TRABECULECTOMIA

Todas as cirurgias serão realizadas pelos mesmos cirurgiões (T.S.P., I. A., D.T.D. e M.U.) seguindo a mesma técnica padronizada, sob anestesia tópica ou peribulbar associada a sedação, sendo que o cirurgião T.S.P. irá participar de todos os procedimentos afim de garantir homogeneidade da técnica. Inicialmente se faz uma corneopexia para exposição do sítio cirúrgico, seguido de peritomia límbica e confecção do flap conjuntival base-fórnice, através da dissecção subtenoniana e hemostasia concomitante. Em seguida, se confecciona um flap escleral de metade da espessura escleral, retangular, medindo aproximadamente 4x2mm na região superior e aplica-se mitomicina C 0,33mg/ml subtenoniano com o uso de esponjas o mais posteriormente possível na região conjuntival, durante 3 minutos. Após os 3 minutos, removem-se as esponjas e procede-se a lavagem com 10 ml de solução salina balanceada e realiza-se uma paracentese acessória. O próximo passo consiste na remoção de uma área de 1x1 mm de tecido corneal periférico sob o flap – trabeculectomia – e iridectomia periférica. Procede-se com a sutura do flap escleral com nylon 10.0 de modo a se observar um fluxo satisfatório de humor aquoso associado a uma boa tensão do globo ocular e manutenção de câmara anterior formada (geralmente 2 a 3 suturas separadas; suturas removíveis serão utilizadas sempre que necessário). E, por fim, sutura-se a conjuntiva com nylon 10.0 com pontos separados. A única diferença entre os grupos será a injeção de acetato de triancinolona subtenoniano (4 mg – 0,1 ml; Ophthalmos Indústria Farmacêutica) ao final da cirurgia na região da ampola filtrante criada, no grupo de intervenção.

O regime de colírios pós operatórios para todos os pacientes incluirá antibioticoprofilaxia com quinolona de 4ª geração 4 vezes ao dia por 7 dias, atropina 1% 2 vezes ao dia por 14 dias e acetato de prednisolona 0,1% inicialmente de 2 em 2 horas, sendo essa dose reduzida de acordo com os níveis de inflamação conjuntival apresentados pelo paciente durante o pós operatório.

A realização de remoção de suturas será realizada de acordo com os níveis pressóricos apresentados pelo paciente no pós operatório a critério médico. Quando, após a lise de suturas, o funcionamento da trabeculectomia não estiver sendo adequado o suficiente para alcançar a PIO alvo estabelecida no pré operatório para cada paciente, em duas avaliações consecutivas, medidas adicionais poderão ser necessárias. Inicialmente, será realizada a reintrodução de medicações hipotensoras tópicas de modo escalonado (maleato de timolol seguido por cloridrato de dorzolamida ou tartarato de brimonidina – desde que sem contraindicações), e, caso mantenha-se a PIO fora do alvo pré estabelecido, será realizado agulhamento. Esse mesma sequência de tratamento pode ser repetida uma vez, se necessário. Caso a PIO novamente mantenha-se fora do alvo estabelecido no pré-operatório após o segundo agulhamento, será feita a reintrodução de medicações hipotensoras tópicas de modo escalonado (maleato de timolol seguido por cloridrato de dorzolamida ou tartarato de brimonidina e, por último análogo de prostaglandina - desde que sem contraindicações). Nessa situação, caso o insucesso no controle da PIO se mantenha, será realizada nova cirurgia antiglaucomatosa (nesse caso, considera-se reoperação).

- 1. DESFECHOS E DEFINIÇÕES DE SUCESSO E FALÊNCIA

Os desfechos principais avaliados serão: diferenças entre os valores de PIO e número de medicações ao longo do pós-operatório e taxas de sucesso entre os grupos. Serão também analisados: complicações intra e pós-operatórias e necessidade de agulhamento ou reoperação.

Serão utilizados 2 critérios de sucesso: PIO ≤ 18mmHg (critério1) e PIO ≤ 15mmHg (critério 2). Quando o sucesso for alcançado sem necessidade de medicações hipotensoras, será considerado sucesso completo e quando alcançado com ou sem a introdução de medicações hipotensoras, será classificado como sucesso qualificado.

Falência será definida através da presença de um dos seguintes parâmetros: hipotonia ocular com repercussão clinica (desde que não revertida em até 3 meses do evento), perda de percepção luminosa, PIO que não preencha os critérios de sucesso em 2 vistas consecutivas e necessidade de nova cirurgia para controle da PIO. A necessidade de agulhamento nos 6 primeiros meses não foi considerada como critério de falência, sendo considerada como manobra ainda relacionada ao pós operatório. Agulhamento foi considerado como reintervenção cirúrgica e, portanto, como falência, quando realizado após 6 meses em pacientes que não alcançaram a PIO alvo estabelecida no pré-operatório após reintrodução de 3 classes de medicações hipotensoras.

- 1. ANÁLISE DOS DADOS

Dados clínicos e demográficos serão apresentados de forma descritiva. O teste de D’Agostino Pearson será realizado para determinar quais variáveis seguem a distribuição normal. Variáveis que sigam distribuição normal serão apresentadas através de média e desvio padrão, enquanto aquelas que não sigam a distribuição normal serão apresentadas através de mediana e intervalos interquartis. As comparações entre os grupos para as variáveis contínuas que sigam distribuição normal serão realizadas através de teste *t,* enquanto aquelas que não sigam a distribuição normal serão analisadas pelo teste de Mann-Whitney. Teste exato de Fischer ou Chi-quadrado será utilizado para avaliar varáveis categóricas quando apropriado. Análise de sobrevivência de Kaplan-Meier e teste de log-rank serão utilizados para deteminar e comparar as taxas de sucesso em cada grupo ao longo do tempo. Os dados serão processados de modo computadorizado através do *software* Medcalc (MedCalc Inc., Mariakerke, Bélgica), sendo adotado *P*<0,05 como nível de significância estatística.

1. CRONOGRAMA

| ETAPAS | 2017 | | | |
| --- | --- | --- | --- | --- |
|  | SET | OUT | NOV | DEZ |
| Definição do tema | X |  |  |  |
| Pesquisa bibliográfica | X | X | X | X |
| Elaboração do Projeto | X |  |  |  |
| Encaminhamento ao CEP | X | X |  |  |
| Coleta de dados |  | X | X | X |

| ETAPAS | 2018 | | | | | | | | | | |
| --- | --- | --- | --- | --- | --- | --- | --- | --- | --- | --- | --- |
|  | JAN | FEV | MAR | ABR | JUN | JUL | AGO | SET | OUT | NOV | DEZ |
| Pesquisa bibliográfica | X | X | X | X | X | X | X | X | X | X | X |
| Coleta de dados | X | X | X | X | X | X | X | X | X | X | X |

| ETAPAS | 2019 | | | | | | | | | | |
| --- | --- | --- | --- | --- | --- | --- | --- | --- | --- | --- | --- |
|  | JAN | FEV | MAR | ABR | JUN | JUL | AGO | SET | OUT | NOV | DEZ |
| Pesquisa bibliográfica | X | X | X | X | X | X | X | X | X | X | X |
| Coleta de dados | X | X | X | X | X | X | X | X | X | X | X |

| ETAPAS | 2020 | | | | | | | |
| --- | --- | --- | --- | --- | --- | --- | --- | --- |
|  | JAN | FEV | MAR | ABR | JUN | JUL | AGO | SET |
| Pesquisa bibliográfica | X | X | X |  |  |  |  |  |
| Coleta de dados | X | X |  |  |  |  |  |  |
| Análise dos dados |  | X | X |  |  |  |  |  |
| Revisão final |  |  | X |  |  |  |  |  |
| Apresentação |  |  | X |  |  |  |  |  |

1. ORÇAMENTO

|  | Valor (R$)  (Unidade) | Quantidade Necessária | Valor (R$) (Total) |
| --- | --- | --- | --- |
| 1. LEVANTAMENTO BIBLIOGRÁFICO |  |  |  |
| 1.1 Artigos científicos | 80,00 | 10 | 800,00 |
| 1.2 Fotocópia | 0,50 | 80 | 40,00 |
|  |  |  |  |
| 2. APRESENTAÇÃO |  |  |  |
| 2.1 Resma de papel A4 | 50,00 | 1 | 50,00 |
| 2.2 Cartucho de tinta | 50,00 | 2 | 100,00 |
| 2.3 Encadernação | 3,00 | 5 | 15,00 |
|  |  |  |  |
| Total |  |  | 1.005,00 |

1. REFERÊNCIAS BIBLIOGRÁFICAS

1. Kingman S. Glaucoma is second leading cause of blindness globally. *Bulletin of the World Health Organization* 2004; **82**(11): 887-8.

2. Tham YC, Li X, Wong TY, Quigley HA, Aung T, Cheng CY. Global prevalence of glaucoma and projections of glaucoma burden through 2040: a systematic review and meta-analysis. *Ophthalmology* 2014; **121**(11): 2081-90.

3. Feiner L, Piltz-Seymour JR. Collaborative Initial Glaucoma Treatment Study: a summary of results to date. *Current opinion in ophthalmology* 2003; **14**(2): 106-11.

4. Lichter PR, Musch DC, Gillespie BW, et al. Interim clinical outcomes in the Collaborative Initial Glaucoma Treatment Study comparing initial treatment randomized to medications or surgery. *Ophthalmology* 2001; **108**(11): 1943-53.

5. Schwartz K, Budenz D. Current management of glaucoma. *Current opinion in ophthalmology* 2004; **15**(2): 119-26.

6. Hosseini H, Mehryar M, Farvardin M. Focus on triamcinolone acetonide as an adjunct to glaucoma filtration surgery. *Medical hypotheses* 2007; **68**(2): 401-3.

7. Lama PJ, Fechtner RD. Antifibrotics and wound healing in glaucoma surgery. *Survey of ophthalmology* 2003; **48**(3): 314-46.

8. Jones E, Clarke J, Khaw PT. Recent advances in trabeculectomy technique. *Current opinion in ophthalmology* 2005; **16**(2): 107-13.

9. Hogewind BF, Pijl B, Hoyng CB, Theelen T. Purified triamcinolone acetonide as antifibrotic adjunct in glaucoma filtering surgery. *Graefe's archive for clinical and experimental ophthalmology = Albrecht von Graefes Archiv fur klinische und experimentelle Ophthalmologie* 2013; **251**(4): 1213-8.

10. Cheung JC, Wright MM, Murali S, Pederson JE. Intermediate-term outcome of variable dose mitomycin C filtering surgery. *Ophthalmology* 1997; **104**(1): 143-9.

11. Matsuda T, Tanihara H, Hangai M, Chihara E, Honda Y. Surgical results and complications of trabeculectomy with intraoperative application of mitomycin C. *Japanese journal of ophthalmology* 1996; **40**(4): 526-32.

12. Perkins TW, Gangnon R, Ladd W, Kaufman PL, Heatley GA. Trabeculectomy with mitomycin C: intermediate-term results. *Journal of glaucoma* 1998; **7**(4): 230-6.

13. Araujo SV, Spaeth GL, Roth SM, Starita RJ. A ten-year follow-up on a prospective, randomized trial of postoperative corticosteroids after trabeculectomy. *Ophthalmology* 1995; **102**(12): 1753-9.

14. Roth SM, Spaeth GL, Starita RJ, Birbillis EM, Steinmann WC. The effects of postoperative corticosteroids on trabeculectomy and the clinical course of glaucoma: five-year follow-up study. *Ophthalmic surgery* 1991; **22**(12): 724-9.

15. Starita RJ, Fellman RL, Spaeth GL, Poryzees EM, Greenidge KC, Traverso CE. Short- and long-term effects of postoperative corticosteroids on trabeculectomy. *Ophthalmology* 1985; **92**(7): 938-46.

16. Anand N, Arora S, Clowes M. Mitomycin C augmented glaucoma surgery: evolution of filtering bleb avascularity, transconjunctival oozing, and leaks. *The British journal of ophthalmology* 2006; **90**(2): 175-80.

17. Ferrante P, Ramsey A, Bunce C, Lightman S. Clinical trial to compare efficacy and side-effects of injection of posterior sub-Tenon triamcinolone versus orbital floor methylprednisolone in the management of posterior uveitis. *Clinical & experimental ophthalmology* 2004; **32**(6): 563-8.

18. Bonini-Filho MA, Jorge R, Barbosa JC, Calucci D, Cardillo JA, Costa RA. Intravitreal injection versus sub-Tenon's infusion of triamcinolone acetonide for refractory diabetic macular edema: a randomized clinical trial. *Investigative ophthalmology & visual science* 2005; **46**(10): 3845-9.

19. Asano S, Miyake K, Miyake S, Ota I. Relationship between blood-aqueous barrier disruption and ischemic macular edema in patients with branch or central retinal vein occlusion: effects of sub-tenon triamcinolone acetonide injection. *Journal of ocular pharmacology and therapeutics : the official journal of the Association for Ocular Pharmacology and Therapeutics* 2007; **23**(6): 577-84.

20. Giangiacomo J, Dueker DK, Adelstein E. The effect of preoperative subconjunctival triamcinolone administration on glaucoma filtration. I. Trabeculectomy following subconjunctival triamcinolone. *Archives of ophthalmology (Chicago, Ill : 1960)* 1986; **104**(6): 838-41.

21. Kahook MY, Camejo L, Noecker RJ. Trabeculectomy with intraoperative retrobulbar triamcinolone acetonide. *Clinical ophthalmology (Auckland, NZ)* 2009; **3**: 29-31.

22. Tham CC, Li FC, Leung DY, et al. Intrableb triamcinolone acetonide injection after bleb-forming filtration surgery (trabeculectomy, phacotrabeculectomy, and trabeculectomy revision by needling): a pilot study. *Eye (London, England)* 2006; **20**(12): 1484-6.

23. Yuki K, Shiba D, Kimura I, Ohtake Y, Tsubota K. Trabeculectomy with or without intraoperative sub-tenon injection of triamcinolone acetonide in treating secondary glaucoma. *American journal of ophthalmology* 2009; **147**(6): 1055-60, 60.e1-2.

24. Foster PJ, Buhrmann R, Quigley HA, Johnson GJ. The definition and classification of glaucoma in prevalence surveys. *The British journal of ophthalmology* 2002; **86**(2): 238-42.

25. Swanson MW. The 97.5th and 99.5th percentile of vertical cup disc ratio in the United States. *Optometry and vision science : official publication of the American Academy of Optometry* 2011; **88**(1): 86-92.
