# Supplementary material for: Subtenon triamcinolone as an adjuvant in mitomycin-C-enhanced trabeculectomy in non-inflammatory glaucomas: A randomized clinical trial
Source: PLoS One. 2022 May 26;17(5):e0268623. doi: 10.1371/journal.pone.0268623 (PMC9135266; doi:10.1371/journal.pone.0268623)
Supplement: S3 File — This file presents the main points of protocol submitted for approval to the ethics committee. (DOC) [file pone.0268623.s003.doc]

**RESEARCH TITLE:**

**SUBTENON TRIAMCINOLONE AS AN ADJUVANT IN MITOMYCIN-C-ENHANCED TRABECULECTOMY IN NON-INFLAMMATORY GLAUCOMAS: A RANDOMIZED CLINICAL TRIAL**

**RESEARCH TEAM:**

Diego Torres Dias, MD; Izabela Almeida, MD; Michele Ushida, MD; Flavio Siqueira Lopes, MD; Fabio Nishimura Kanadani, MD, PhD; Tiago Santos Prata*, MD, PhD

*t.prata0807@gmail.com

**RESEARCH INSTITUTION:**

Glaucoma Sector – Department of Ophthalmology – Federal University of São Paulo

September, 2017

**SUMMARY**

Trabeculectomy has been the most widely employed incisional surgery technique for glaucoma treatment worldwide. Nonetheless, surgical success depends on controlled inhibition of the cicatrization process. This trial was designed to assess the adjuvant effect of subtenon triamcinolone acetonide (TAAC) in surgical success rates of primary trabeculectomies (TRABs) in individuals with non-inflammatory glaucomas. This is an unicentric, non-masked, randomized clinical trial. Consecutive patients with clinically uncontrolled non-inflammatory glaucomas and indication of TRAB surgery will be recruited between January 2017 and February 2018. The individuals will be randomly enrolled to one of the two study groups and, therefore, were submitted either to mytomycin(MMC)-enhanced TRAB or MMC-enhanced TRAB with an adjuvant injection of subtenon TAAC at the end of surgery, respectively. Intraocular pressure (IOP) and number of IOP-lowering medications will be assessed preoperatively and follow-up visits will be scheduled for 1, 3, 6, 12, 18 and 24 months post-op. Statistical significance was set at P<0.05 and computerized analysis will be performed using MedCalc software (MedCalc, Inc., Mariakerke, Belgium).

1. **RATIONALE AND BACKGROUND INFORMATION**

Glaucoma is the main cause of irreversible blindness worldwide.^1^ It is estimated that approximately 64.3 million people between 40 and 80 years old are currently affected by the disease, and this number may rise up to 76 million, in 2020, and 111.8 million, in 2040.^2^ Initial treatment for these patients is most frequently started clinically with hypotensive eyedrops, while incisional surgeries are often employed in medically uncontrolled cases, which did not respond to maximum tolerated topical and laser therapy.^3,4^

more than 20 years, trabeculectomy (TRAB) has been the most widely employed incisional surgery technique for glaucoma treatment worldwide.^5^ Nonetheless, surgical success depends on controlled inhibition of the cicatrization process.^6,7^ In this context, surgical failure is most frequently associated with excessive formation of scar tissue in the subtenon space that prevents adequate aqueous outflow through the created fistula.^8,9^ As an approach to address this issue, modulation of wound healing measures, such as the intraoperative use of antifibrotic drugs (the most widely employed is mitomycin C – MMC)^10-12^ and postoperative topical corticosteroids^13-15^ are routinely employed in glaucoma surgery, as they improve success rates in TRABs.

Corticosteroid eyedrops have been for a long time paramount for wound healing control as a postoperative therapy in TRABs. Additionally, subtenon injection of triamcinolone acetonide (TAAC) is not only a safe and effective ophthalmic procedure, but also a well-established therapeutic option for several ophthalmic conditions (such as posterior uveitis,^16^ diabetic macular edema^17^ and macular edema secondary to vascular occlusions^18^). With that in mind, one might hypothesize that a deposit corticosteroid formulation, such as TAAC, might increase its postoperative bioavailability, and possibly improve wound healing control and surgical outcomes in TRABs.

*STUDY GOALS*

The purpose of this randomized clinical trial was to assess the adjuvant effect of subtenon TAAC injection in surgical outcomes of primary TRABs in individuals with non-inflammatory glaucomas.

1. **STUDY DESIGN AND METHODS**

*ETHICS*

This protocol adhered to the tenets of the declaration of Helsinki. To be included in this study, all patients will have to provide written informed consent prior to enrollment and examination.

*STUDY DESIGN*

This will be an unicentric, randomized for a intended 1:1 allocation ratio, parallel-group, unmasked clinical trial, designed to assess the adjuvant effect of subtenon TAAC in surgical outcomes of primary TRABs in individuals with non-inflammatory glaucomas.

*PARTICIPANTS*

Consecutive glaucomatous patients with medically uncontrolled non-inflammatory glaucomas and indication for TRAB attending to the Glaucoma Sector of Hospital Medicina dos Olhos (Sao Paulo, Brazil) will be enrolled in this study.

For that purpose, glaucoma will be defined as the presence of glaucomatous optic neuropathy (GON) associated or not with characteristic visual field (VF) defect. GON will be defined as cup-to-disc ratio >0.6, asymmetry between eyes ≥0.2, presence of localized defects of the retinal nerve fiber layer, and/ or neuroretinal rim in the absence of any other anomalies that could explain such findings. Non-inflammatory glaucoma will be defined as glaucoma not associated with ocular inflammation (neovascular or glaucoma associated with uveitis). Additionally, characteristic glaucomatous VF defect will be defined as glaucoma hemifield test results outside normal limits and the presence of at least 3 contiguous test points within the same hemifield on the pattern deviation plot at P<1%, with at least 1 at P<0.5%, excluding points on the edge of the field or those directly above and below the blind spot.^19,20^ Finally, a patient will be considered to be medically uncontrolled based on anatomical and/or functional progression (detected by retinography or perimetry, respectively) or IOP values above the target range defined by the attending physician based on VF, optic nerve examination, age and risk factors, in accordance with European guidelines.^21^

Patients will be excluded if they meet one of the following criteria: diagnosis of inflammatory glaucomas (neovascular or glaucoma associated with uveitis); diagnosis of medically uncontrolled non-inflammatory glaucomas associated with clinically relevant cataracts, and indication of combined surgery (phacoemulsification plus TRAB); and previous intraocular surgery, except for uncomplicated phacoemulsification or laser surgery if performed more than 6 months prior to glaucoma surgery.

*PROCEDURES AND SURGICAL TECHNIQUE*

All participants will be submitted to a comprehensive ophthalmological evaluation, including best-corrected visual acuity, slit-lamp biomicroscopy, IOP measurement, gonioscopy, dilated fundoscopy, VF testing (24-2 Swedish interactive threshold algorithm, Humphrey Field Analyzer II; Carl Zeiss Meditec, Inc., Dublin, CA), optic disc stereophotographs, and color/ red-free fundus imaging. Clinical and ocular data including age, race, gender, IOP, central corneal thickness (CCT), VF mean deviation (MD), previous intraocular surgery and type of glaucoma were assessed at the baseline visit. Follow-up visits will be scheduled at 1, 3, 6, 12, 18 and 24 months postoperatively. Additional follow-up visits will be performed if necessary.

After inclusion, patients will be randomly enrolled in two groups by flip-coin technique, performed by the chief-nurse at the operating room immediately before surgery. In the control group, eyes will be submitted to a standard MMC-enhanced TRAB (MMC-TRAB), while in the intervention group, besides the standard MMC-TRAB, these eyes will also be submitted to a subtenon TAAC injection (4mg) close to the bleb site at the end of the surgery.

All surgeries will be performed by 4 surgeons with previous glaucoma surgery experience (T.S.P., D.T.D., I.A., and M.U.), following a standard technique under peribulbar anesthesia and sedation. In order to maintain homogeneity of the surgical technique, T.S.P. will be present in all of the procedures. Initially, a traction corneal suture will be performed to allow better exposure of the surgical site. This will be followed by incision of the conjunctiva and Tenon’s capsule 1-2mm posterior to the limbus and fornix-based subtenon dissection posteriorly in the superior quadrant, with concomitant hemostasis as needed. MMC at the concentration of 0.33 mg/ml will then be applied for 3 minutes under the Tenon’s capsule using 3 separate soaked sponges. This will be followed by dissection of a rectangular 4x2mm half-thickness scleral ﬂap up to the clear cornea using a crescent knife, resection of an anterior trabecular block and peripheral iridectomy. The scleral ﬂap will then be sutured with two or three 10-0 nylon sutures, and the suture tension will be adjusted to allow adequate ﬂow of aqueous and at the same time maintain anterior chamber depth. Finally, the conjunctiva and Tenon’s capsule will be closed with separate 10-0 nylon sutures. The only difference between groups will be the subtenon TAAC injection (4 mg – 0.1 ml; Ophthalmos Pharmaceuticals) at the bleb site 7mm posterior to the limbus in the intervention group.

The postoperative eyedrop regimen will follow the same protocol for both groups. All patients will be treated postoperatively with topical antibiotic eyedrops (moxifloxacin) 4 times daily for a week, topical 1% atropine 2 times daily for 2 weeks and topical corticosteroid eyedrops (prednisolone 1%). The topical corticosteroids will be initially applied every 1 hour and this dose was then tapered according to levels of postoperative conjunctival inflammation. Additionally, laser suture lysis will be performed as needed and the introduction of hypotensive eyedrops will be performed in a stepwise protocol^22^ whenever the patient presents at two consecutive appointments with IOP higher than the preoperatively defined target range. Finally, when IOP control is not achieved with the reintroduction of medication, needling will be performed, and, if IOP remain higher than the preoperatively defined target range at two consecutive appointments, hypotensive eyedrops will be reintroduced in a stepwise manner.

*CLINICAL OUTCOMES AND DEFINITIONS OF SUCCESS*

The main outcome of this clinical trial will be the difference in IOP between groups at the 24-month follow-up. Secondary outcomes will be difference in postoperative success rates and number of hypotensive eyedrops between groups at all timepoints.

In accordance with the World Glaucoma Association Guidelines on Design and Reporting Clinical Trials,^23^ we will define success as IOP ≤ 15mmHg. Whenever success is achieved without the need for additional hypotensive medications, this will be considered complete success. On the other hand, whenever the IOP-based criterion is achieved only after the introduction of hypotensive eyedrops, this will be considered qualified success.

Additionally, surgical failure will be defined as the presence of one of the following criteria: loss of light perception, IOP that does not fulfill the success criteria in 2 consecutive follow-up visits and IOP-lowering reoperations. Eyes submitted to needling procedures or surgical approaches due to overfiltration (such as conjunctival restrictive sutures or choroidal drainage) during the first 6 months of follow-up will not be considered surgical failures, since these are considered early postoperative-related maneuvers. Nonetheless, whenever these procedures become necessary after 6 months of follow-up, they will be labeled as reoperations and, therefore, surgical failures.

Needling and reoperations rates and surgical complications, such as hyphema, hypotonic maculopathy, choroidal detachment and persistent bleb leakage will also be evaluated at the same timepoints. In this context, a bleb leakage will be considered as persistent whenever it does not resolve spontaneously after 1 month with conservative medical management or requires surgical reintervention.^24^

*SAMPLING AND STATISTICAL ANALYSIS*

Considering the primary outcome of our study, we chose the magnitude of IOP reduction difference between groups at 24 months of follow-up as the main variable for sample size calculation. For a sample power of 80% (β value of 0.20) and α value (type I error) of 0.05, we will need 29 patients in each group to detect an IOP difference of 3 mmHg between groups at the 2-year follow-up (assuming a standard deviation of 4 mmHg).

The analysis of our results will be based on initial randomization (Intent to Treat Analysis). Descriptive analysis will be used to present demographic and clinical data. D’Agostino-Pearson test will be performed to determine whether data had normal distribution. Normally-distributed data will be presented as mean and standard deviation, whether non normally-distributed data will be presented as median and interquartile intervals. Regarding the comparison between groups, continuous data will be compared using t test or Mann-Whitney test, depending on the data distribution. Fischer’s exact test or Chi-square test will be performed to evaluate categorical variables whenever appropriate. Kaplan-Meier survival analysis and log-rank test will be used to estimate and compare success rates between groups along the postoperative follow-up period. All included eyes will be considered for the Kaplan-Meier survival analysis. Computerized analysis will be performed using MedCalc software (MedCalc Inc., Mariakerke, Belgium) and statistical signiﬁcance will be set at P<0.05.

1. **PROJECT TIMELINE**

|  | 2017 | | | | | | | | | | | |
| --- | --- | --- | --- | --- | --- | --- | --- | --- | --- | --- | --- | --- |
|  | JAN | FEB | MAR | APR | MAY | JUN | JUL | AUG | SEP | OCT | NOV | DEC |
| Project Elaboration |  |  |  |  |  |  |  |  | X | X | X | X |
| Bibliographic review X X X X X X X X X X X X |  |  |  |  |  |  |  |  | X | X |  |  |
| Submission to Ethics Comitee |  |  |  |  |  |  |  |  | X | X | X |  |
| Data Collection |  |  |  |  |  |  |  |  |  |  | X | X |

|  | 2018 | | | | | | | | | | | |
| --- | --- | --- | --- | --- | --- | --- | --- | --- | --- | --- | --- | --- |
|  | JAN | FEB | MAR | APR | MAY | JUN | JUL | AUG | SEP | OCT | NOV | DEC |
| Bibliographic review | X | X | X | X | X | X | X | X | X | X | X | X |
| Data Collection | X | X | X | X | X | X | X | X | X | X | X | X |

|  | 2019 | | | | | | | | | | | |
| --- | --- | --- | --- | --- | --- | --- | --- | --- | --- | --- | --- | --- |
|  | JAN | FEB | MAR | APR | MAY | JUN | JUL | AUG | SEP | OCT | NOV | DEC |
| Bibliographic review | X | X | X | X | X | X | X | X | X | X | X | X |
| Data Collection | X | X | X | X | X | X | X | X | X | X | X |  |

|  | 2020 | | | | | | | | | | | |
| --- | --- | --- | --- | --- | --- | --- | --- | --- | --- | --- | --- | --- |
|  | JAN | FEB | MAR | APR | MAY | JUN | JUL | AUG | SEP | OCT | NOV | DEC |
| Bibliographic review | X | X | X |  |  |  |  |  |  |  |  |  |
| Data Collection | X | X | X |  |  |  |  |  |  |  |  |  |
| Data Analysis |  | X | X |  |  |  |  |  |  |  |  |  |
| Manuscript Preparation |  |  | X |  |  |  |  |  |  |  |  |  |
| Manuscript Revision |  |  | X |  |  |  |  |  |  |  |  |  |
| Manuscript Submission |  |  |  | X |  |  |  |  |  |  |  |  |

1. **REFERENCES**

1 Kingman, S. Glaucoma is second leading cause of blindness globally. *Bulletin of the World Health Organization* **82**, 887-888, doi:/S0042-96862004001100019 (2004).

2 Tham, Y. C. *et al.* Global prevalence of glaucoma and projections of glaucoma burden through 2040: a systematic review and meta-analysis. *Ophthalmology* **121**, 2081-2090, doi:10.1016/j.ophtha.2014.05.013 (2014).

3 Feiner, L., Piltz-Seymour, J. R. & Collaborative Initial Glaucoma Treatment, S. Collaborative Initial Glaucoma Treatment Study: a summary of results to date. *Current opinion in ophthalmology* **14**, 106-111, doi:10.1097/00055735-200304000-00010 (2003).

4 Lichter, P. R. *et al.* Interim clinical outcomes in the Collaborative Initial Glaucoma Treatment Study comparing initial treatment randomized to medications or surgery. *Ophthalmology* **108**, 1943-1953, doi:10.1016/s0161-6420(01)00873-9 (2001).

5 Schwartz, K. & Budenz, D. Current management of glaucoma. *Current opinion in ophthalmology* **15**, 119-126, doi:10.1097/00055735-200404000-00011 (2004).

6 Hosseini, H., Mehryar, M. & Farvardin, M. Focus on triamcinolone acetonide as an adjunct to glaucoma filtration surgery. *Medical hypotheses* **68**, 401-403, doi:10.1016/j.mehy.2006.04.075 (2007).

7 Lama, P. J. & Fechtner, R. D. Antifibrotics and wound healing in glaucoma surgery. *Survey of ophthalmology* **48**, 314-346, doi:10.1016/s0039-6257(03)00038-9 (2003).

8 Hogewind, B. F., Pijl, B., Hoyng, C. B. & Theelen, T. Purified triamcinolone acetonide as antifibrotic adjunct in glaucoma filtering surgery. *Graefe's archive for clinical and experimental ophthalmology = Albrecht von Graefes Archiv fur klinische und experimentelle Ophthalmologie* **251**, 1213-1218, doi:10.1007/s00417-012-2161-y (2013).

9 Jones, E., Clarke, J. & Khaw, P. T. Recent advances in trabeculectomy technique. *Current opinion in ophthalmology* **16**, 107-113, doi:10.1097/01.icu.0000156138.05323.6f (2005).

10 Cheung, J. C., Wright, M. M., Murali, S. & Pederson, J. E. Intermediate-term outcome of variable dose mitomycin C filtering surgery. *Ophthalmology* **104**, 143-149, doi:10.1016/s0161-6420(97)30347-9 (1997).

11 Matsuda, T., Tanihara, H., Hangai, M., Chihara, E. & Honda, Y. Surgical results and complications of trabeculectomy with intraoperative application of mitomycin C. *Japanese journal of ophthalmology* **40**, 526-532 (1996).

12 Perkins, T. W., Gangnon, R., Ladd, W., Kaufman, P. L. & Heatley, G. A. Trabeculectomy with mitomycin C: intermediate-term results. *Journal of glaucoma* **7**, 230-236 (1998).

13 Araujo, S. V., Spaeth, G. L., Roth, S. M. & Starita, R. J. A ten-year follow-up on a prospective, randomized trial of postoperative corticosteroids after trabeculectomy. *Ophthalmology* **102**, 1753-1759, doi:10.1016/s0161-6420(95)30797-x (1995).

14 Roth, S. M., Spaeth, G. L., Starita, R. J., Birbillis, E. M. & Steinmann, W. C. The effects of postoperative corticosteroids on trabeculectomy and the clinical course of glaucoma: five-year follow-up study. *Ophthalmic surgery* **22**, 724-729 (1991).

15 Starita, R. J. *et al.* Short- and long-term effects of postoperative corticosteroids on trabeculectomy. *Ophthalmology* **92**, 938-946, doi:10.1016/s0161-6420(85)33931-3 (1985).

16 Ferrante, P., Ramsey, A., Bunce, C. & Lightman, S. Clinical trial to compare efficacy and side-effects of injection of posterior sub-Tenon triamcinolone versus orbital floor methylprednisolone in the management of posterior uveitis. *Clinical & experimental ophthalmology* **32**, 563-568, doi:10.1111/j.1442-9071.2004.00902.x (2004).

17 Bonini-Filho, M. A. *et al.* Intravitreal injection versus sub-Tenon's infusion of triamcinolone acetonide for refractory diabetic macular edema: a randomized clinical trial. *Investigative ophthalmology & visual science* **46**, 3845-3849, doi:10.1167/iovs.05-0297 (2005).

18 Asano, S., Miyake, K., Miyake, S. & Ota, I. Relationship between blood-aqueous barrier disruption and ischemic macular edema in patients with branch or central retinal vein occlusion: effects of sub-tenon triamcinolone acetonide injection. *Journal of ocular pharmacology and therapeutics : the official journal of the Association for Ocular Pharmacology and Therapeutics* **23**, 577-584, doi:10.1089/jop.2007.0057 (2007).

19 Foster, P. J., Buhrmann, R., Quigley, H. A. & Johnson, G. J. The definition and classification of glaucoma in prevalence surveys. *The British journal of ophthalmology* **86**, 238-242, doi:10.1136/bjo.86.2.238 (2002).

20 Swanson, M. W. The 97.5th and 99.5th percentile of vertical cup disc ratio in the United States. *Optometry and vision science : official publication of the American Academy of Optometry* **88**, 86-92, doi:10.1097/OPX.0b013e3181fc3638 (2011).

21 European Glaucoma Society Terminology and Guidelines for Glaucoma, 4th Edition - Chapter 3: Treatment principles and options Supported by the EGS Foundation: Part 1: Foreword; Introduction; Glossary; Chapter 3 Treatment principles and options. *The British journal of ophthalmology* **101**, 130-195, doi:10.1136/bjophthalmol-2016-EGSguideline.003 (2017).

22 Fea, A. M. Phacoemulsification versus phacoemulsification with micro-bypass stent implantation in primary open-angle glaucoma: randomized double-masked clinical trial. *Journal of cataract and refractive surgery* **36**, 407-412, doi:10.1016/j.jcrs.2009.10.031 (2010).

23 Association., W. G. Guidelines on Design and Reporting of Glaucoma Surgical Trials. In: Shaarawy TM, Sherwood MB, Grehn F,. *eds. Amsterdam: Kugler Publications;* (2009.).

24 Wang, Q. & Harasymowycz, P. Collagen Cross-linking for Late-onset Bleb Leakage: 1-Year Results. *Journal of glaucoma* **25**, e273-276, doi:10.1097/IJG.0000000000000295 (2016).

1. **FUNDING**

None
