# Supplementary material for: Subtenon triamcinolone as an adjuvant in mitomycin-C-enhanced trabeculectomy in non-inflammatory glaucomas: A randomized clinical trial
Source: PLoS One. 2022 May 26;17(5):e0268623. doi: 10.1371/journal.pone.0268623 (PMC9135266; doi:10.1371/journal.pone.0268623)
Supplement: S5 File — This file presents surgical success data of each surgeon in the study. (DOC) [file pone.0268623.s005.doc]

**Number of eyes operated and success rates** by surgeon

|  | **Surgeon 1 (n= 20)** | **Surgeon 2 (n=22)** | **Surgeon 3 (n=15)** | **Surgeon 4 (n=18)** |
| --- | --- | --- | --- | --- |
| Intervention Group | 11 | 10 | 8 | 10 |
| Control Group | 9 | 11 | 8 | 8 |
| Total complete success surgeries at 24 months (n / %) | 15 (75%) | 16 (72.7%) | 12 (68.1%) | 13 (72.2%) |
| Complete success surgeries in Intervention Group at 24 months (n / %) | 9 (81.8%) | 8 (80%) | 7 (87.5%) | 8 (80%) |
| Complete success surgeries in Control Group at 24 months (n / %) | 6 (66.6%) | 8 (72.7%) | 5 (62.5%) | 5 (62.5%) |
